# Onboard recordings reveal how bats maneuver under severe acoustic interference

**DOI:** 10.1073/pnas.2407810122

**Published:** 2025-03-31

**Authors:** Aya Goldshtein, Omer Mazar, Lee Harten, Eran Amichai, Reut Assa, Anat Levi, Yotam Orchan, Sivan Toledo, Ran Nathan, Yossi Yovel

**Affiliations:** ^a^Centre for the Advanced Study of Collective Behaviour, University of Konstanz, Konstanz 78464, Germany; ^b^Department of Collective Behaviour, Max Planck Institute of Animal Behavior, Konstanz 78464, Germany; ^c^Department of Biology, University of Konstanz, Konstanz 78464, Germany; ^d^Faculty of Life Sciences, School of Zoology, Tel Aviv University, Tel-Aviv 6997801, Israel; ^e^Sagol School of Neuroscience, Tel Aviv University, Tel-Aviv 6997801, Israel; ^f^Department of Ecology, Evolution and Behavior, The Hebrew University of Jerusalem, Jerusalem 9190401, Israel; ^g^Minerva Center for Movement Ecology, The Hebrew University of Jerusalem, Jerusalem 9190401, Israel; ^h^Faculty of Exact Sciences, Blavatnik School of Computer Science, Tel-Aviv University, Tel-Aviv 6997801, Israel; ^i^Faculty of Engineering, School of Mechanical Engineering, Tel Aviv University, Tel-Aviv 6997801, Israel

**Keywords:** emergence, collective behavior, echolocation, collision avoidance, cocktail party nightmare

## Abstract

Echolocating bats rely on active acoustic sensing to perceive their environment. When multiple bats fly together, echolocating simultaneously, the calls emitted by nearby conspecifics could interfere with and mask the echoes necessary for orientation. Nowhere is this impairment of sensing more dramatic than when thousands of bats emerge from a cave at the same time. Here, we tracked the movement of tens of greater mouse-tailed bats flying within a group of thousands. By mounting miniature microphones onboard some of the bats, we monitored the acoustic scene from the point of view of an individual bat within the echolocating collective. We found that bats experienced a very high level of conspecific acoustic masking when emerging from their cave, which dropped within seconds as the bats spread out in space. A comprehensive sensorimotor model, based on the unique data that we collected, revealed how bats content with this severe echo masking almost without collisions. Our results demonstrate that even under severe masking, bats are hardly impaired sensorially, and we suggest how they are able to maneuver smoothly and avoid collisions, even at high densities, without applying a jamming avoidance response.

## Significance

Echolocating bats that actively sense their environment and fly together out of their cave, face a tremendous nightly challenge of maneuvering under severe acoustic interference while trying to avoid collisions. This scenario, which has been termed the “Cocktail Party Nightmare,” was previously examined by placing a microphone near the cave’s entrance. Here, we examined how bats contend with this severe acoustic scenario by tracking the movement of tens of bats simultaneously, while recording the echolocation of some individuals using an onboard miniature microphone, and applying a sensorimotor model. We show how the bats spread out in space rapidly after emerging from their cave and how they manage to reduce the acoustic masking and thereby avoid collisions.

Collective movement is a widespread phenomenon across taxa. To maintain group cohesion while avoiding collisions, individuals must constantly gather information about nearby neighbors and their environment and plan their movement accordingly (1). Most studies examined collective movement under lab conditions and/or highly controlled settings, examining animals relying mainly on vision (2–7), pressure sensing (8, 9), and passive hearing (10), and focusing on their movement algorithms while mostly ignoring the sensory challenge they experience (11–18). Several studies examined sensory aspects of collective movement (6, 19–23), such as which cues are used for maintaining minimal distances between neighbors (23) and how group size and position within the group affect visual detection (2, 6). However, research on the sensory interference faced by individuals in a group is limited.

Bats pose a particularly interesting case of collective movement as they heavily rely on active hearing, i.e., biosonar or echolocation, to coordinate collective movement, maneuver smoothly, and avoid collisions (24). When multiple bats echolocate simultaneously, they could overload the sensory frequency band, masking the weaker echoes returning from nearby conspecifics, which are essential to avoid collisions. Nonetheless, bats somehow manage to handle this “cocktail party nightmare” (25) with very few collisions.

Previous studies on the masking challenge have been mostly performed on bats foraging in small groups (19, 26–30). Some of the strategies suggested for contending with the sensory problem (25), include spectral (26, 27, 31–35) and temporal (36) jamming avoidance, spatial filtering via ear movements (37), marking of self-calls for individual recognition (38), and reliance on spatial memory (25). But note that recent experimental (29, 39–43) and theoretical studies (44, 45) questioned the spectral jamming avoidance hypothesis.

In the typical group-foraging situation, only a few bats fly together, and their density is much lower than when emerging in a group. These bats do not aim to move collectively but rather to catch prey with minimal interindividual interference or to efficiently search for unpredictable and ephemeral food patches (46). Field studies on collective movement in bats are extremely sparse (24, 47) due to the difficulty in monitoring the movement of multiple individuals within the group and even greater difficulty in recording the calls of a single individual within the group.

Here, we overcame these two methodological challenges by applying the ATLAS tracking system (48) to track the movement of tens of greater mouse-tailed bats (*Rhinopoma microphyllum*) emerging from their cave and moving simultaneously within a group of thousands. We also fitted some of the bats with onboard microphones (49), enabling us to record the auditory scene from the individual bat’s point of view. These unique data, complemented by a simple biological-plausible sensorimotor model that probably underestimated the abilities of real bats, enabled us to examine how bats move collectively at such high densities while relying on echolocation. We hypothesized that bats would use a sensorimotor response to reduce the risk of acoustic masking. Accordingly, we predicted that the bats would experience a very high level of masking at the cave exit, decreasing as they move farther away from the cave. Many conspecific echoes would be jammed, but some will not, and this redundancy would enable bats to avoid collisions. We further hypothesized that bats would not apply jamming avoidance response as part of their strategy.

## Results

### Collective Movement Dynamics.

Every evening during summer, within the span of one minute, about 2,000 greater mouse-tailed bats emerges from a small cave opening (~3 m^2^, *Materials and Methods*, Video S1). Greater mouse-tailed bats forage for ephemeral insect swarms whose locations are unknown (41), and thus, the group probably does not have a predefined goal.

**Fig. 1.**
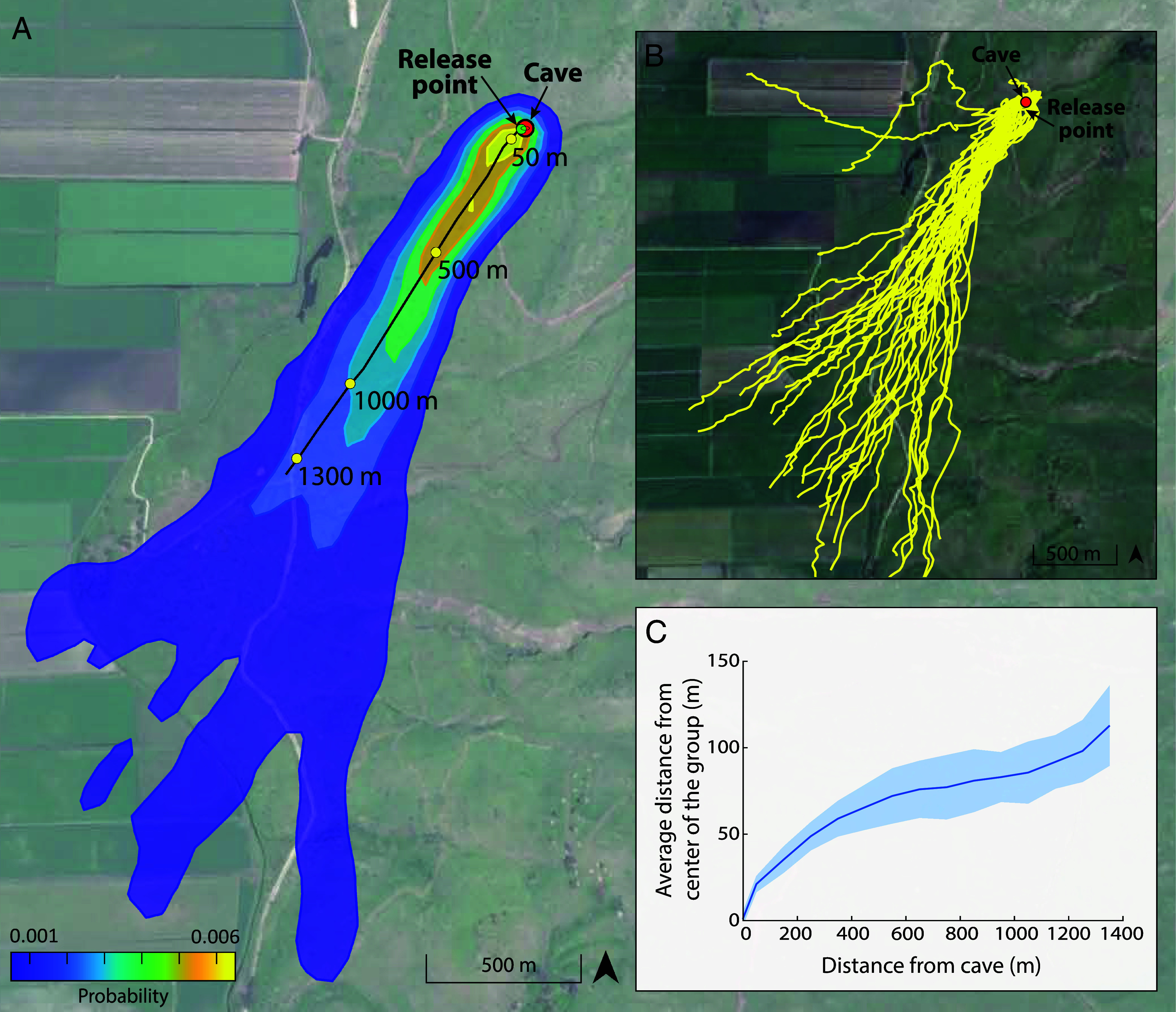
Collective movement dynamics. Tagged bats were individually released at a distance of 20 m from the cave, and immediately joined the natural evening emergence that flew above them to avoid changing the natural density of the group (the cave and the release point are represented by red and green circles, respectively). (*A*) The density of a group of ~2,000 bats during their evening emergence in 2019. Colors represent the 2D bat distribution (*Materials and Methods*). The black line represents the center of the group. (*B*) Monitoring the flight trajectories of 37 bats simultaneously (yellow lines) effectively revealed how most bats (35 bats) emerged from the cave in a group and then gradually increased their distance from the center of the group as a function of flight distance. Two bats quickly abandoned the group and are excluded from the analysis. (*C*) The average distance from the group center as a function of the distance from the cave. Data present all bats that flew in the main group: 75 and 95% out of 59 and 37 bats, with tracking data from 2018 and 2019, respectively.

High-resolution simultaneous tracking of 37 and 59 of these bats in two consecutive years allowed us to uncover the movement patterns within the group. The bats moved collectively for an average distance of ~1.3 km from their cave (Fig. 1 *A* and *B* and *SI Appendix*, Fig. S1). As the bats flew farther from the cave, the group structure widened, spreading over a wide front of up to a kilometer at a distance of 2.5 km from the cave in comparison to a width of only 130 m at 300 m from the cave (the width was defined according to where bat density drops to 50%, Fig. 1 and *SI Appendix*, Fig. S1). The widening of the group resulted in an increase of the average minimal interindividual distance from 14.0 ± 5.7 m at 25 m to 64.2 ± 22.2 m at 300 m away from the cave (n = 30 simulations; *Materials and Methods*). Bats’ flight ground speed gradually increased from 7 to 10.5 m/s during the first 650 m of flight and then remained stable.

### Sensory Masking and Collision Rate.

We next examined how bats contend with severe sensory masking when flying as a collective and whether they are able to rely on echolocation (only) while doing so. To avoid collision, the bats must receive the echoes returning from nearby bats in order to localize their neighbors and plan their movement. These echoes, which are inherently weak due to the double-attenuation of the sound wave, can be missed due to the sensory masking created by the loud calls of conspecifics. The analysis of onboard audio recordings of four bats flying within the group, along with a detailed sensorimotor model, indicated that the fast decrease in bat density (Fig. 1) leads to a rapid decrease in sensory masking and a very low probability of collision.

**Fig. 2.**
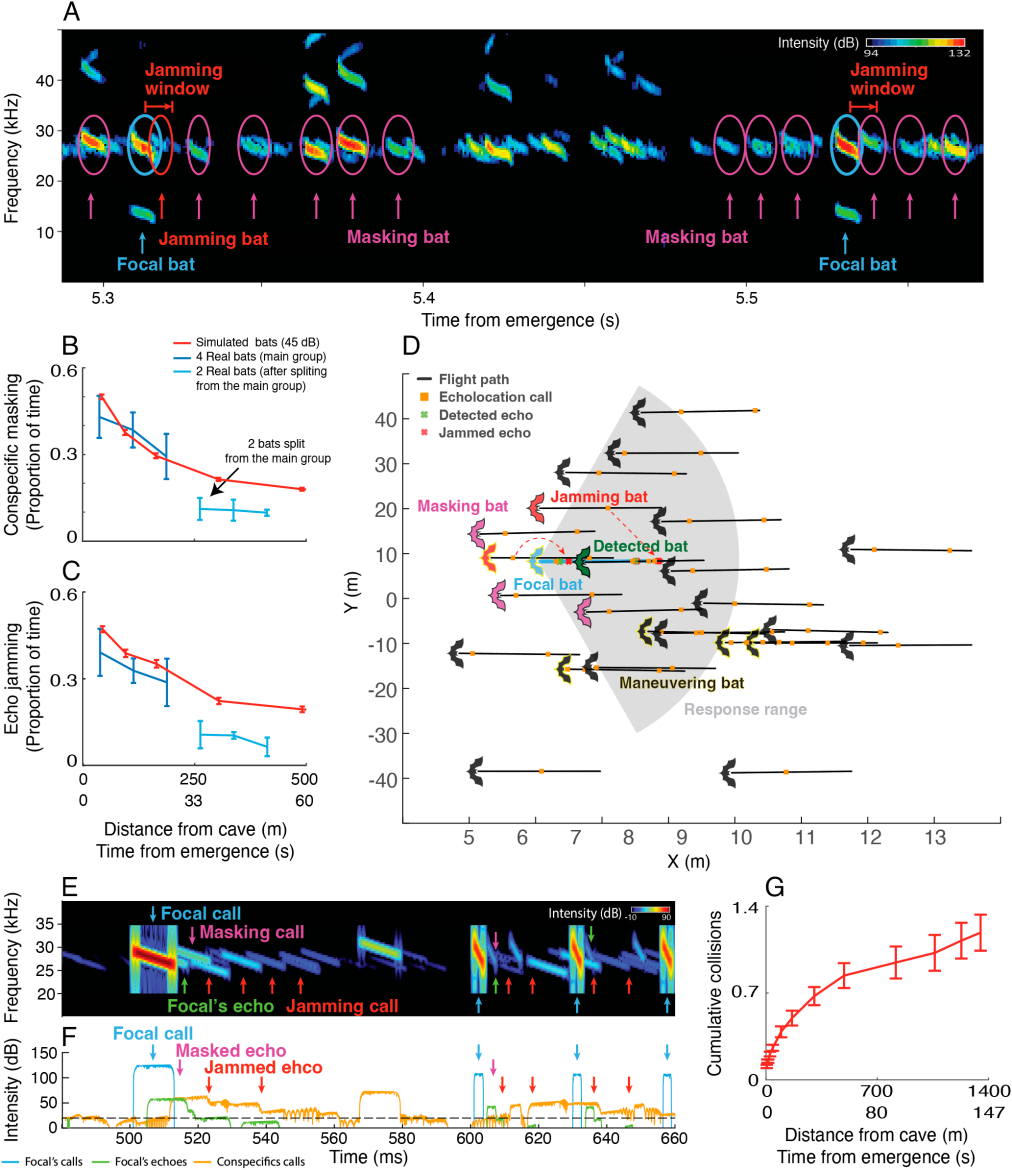
Masking levels and collision rate in the collective structure. (*A*) The acoustic scene was recorded at a distance of ~40 m from the cave by one of the tags attached to a bat. Focal calls, masking, and jamming calls are marked. Jamming calls overlap with potential echoes from nearby conspecifics and are louder than them. (*B* and *C*) Echolocation masking and jamming proportion as a function of flight distance or time from the cave (data represent mean and SE for four real bats (blue) and for 30 simulations for the simulated bats (red). Note that panels *B* and *C* begin at 25 m from the cave because this is where we have data for the real bats. Data for four bats that fly within the main collective are depicted in blue (25 to 200 m from the cave), and data for 2 bats that split from the main collective and fly with a smaller group are depicted in light blue (200 to 450 m from the cave). The red lines in *B* and *C* show the masking-jamming proportions for simulated bats when using a hearing threshold of 45 dB-SPL (the noise-floor of our microphone). (*D*) An illustration of a snapshot of the sensorimotor model. In this simulation, 25 bats fly for 180 ms (black lines), at a distance of 40 m from the cave, emit echolocation (orange square) and respond (maneuver, yellow edge line) according to the reflected echoes to avoid collisions. The gray shaded area depicts the focal bat’s response range at time 0, which is the active echolocation range for detecting a conspecific by the focal bat (3 m with a 60° double-sided sector). The focal bat (blue) emits echolocation calls that are reflected by nearby bats. The echoes of its first echolocation call are reflected from its conspecifics, some of the echoes (red x) are jammed by a conspecific (red bat), but the echo (green x) reflected from the bat in front of it (green bat) is detected, and it responds in shorter and more frequent calls while maneuvering (yellow edge) to avoid collision. (*E*) Spectrogram and (*F*) Oscillogram of the illustrated snapshot of the sensorimotor model as received by the focal bat. (*E*) Echolocation calls of the focal bat (blue arrow) and the echoes it receives from near neighbors are marked (green arrows). Conspecific masking (pink arrows) and jamming (red arrows) calls are marked. (*F*) The intensity of the echolocation calls of the focal bat (blue), its echoes (green), and conspecifics’ echolocation calls (orange) are presented. Some echoes of the echolocation of the focal bat were masked (pink arrows) by weaker conspecifics’ calls or jammed (red arrows) by stronger conspecifics’ calls. The hearing threshold of the bat (20 dB) is marked (black dashed line). (*G*) The cumulative number of collisions per bat as a function of the distance/time from the cave [data represent mean and SE for 30 simulations for the simulated bats (red)]. This low collision rate has been confirmed from multiple videos in which we [and other researchers observed hardly any collision (50)].

Conspecific masking probability (i.e., the proportion of time calls might be masked) decreased rapidly within seconds, decaying from 0.45 ± 0.13 to 0.36 ± 0.2 when bats flew from 25 m to 200 m from their cave within ~25 s (*Materials and Methods*, Fig. 2*A* and *SI Appendix*, Fig. S2 and Fig. 2*B*, blue line).

The echo-jamming (i.e., the proportion of time conspecific echoes would be missed; see *Materials and Methods* and schematic in Fig. 2*A*) also decreased from 0.42 ± 0.1 to 0.39 ± 0.2 when bats flew from a distance of 25 to 200 m away from their cave (Fig. 2*C*, blue line). This analysis only sought to reveal the rapid decrease in jamming over time, while our model, presented below, already incorporates a more sensitive biological-like receiver.

Next, we used a sensorimotor model (44) to simulate bats moving in a group while using echolocation only. The simulated bats were normally distributed within a 2D area according to the observed distribution of real bats (Fig. 1*A*, *Materials and Methods*, Video S1). They used echolocation to sense their surroundings and reacted to the returning echoes by adjusting their echolocation and flight direction to avoid collisions. We used the model to simulate snapshots of the collective movement at 13 distances (between 5 and1,350 m) from the cave, simulating a one-second flight of 25 bats (Fig. 1*C*), and running 30 simulations for each distance (see *Sensorimotor Model* in *Materials and Methods* and Table 1).

**Table 1.** The echolocation parameters in the different phases [Adjusted from (51), frequency values were taken from our on-board recordings]

Flight phase	Search	Approach		Buzz		
Parameter		Start	End	Terminal 1 start	Terminal 1 end	Terminal 2
Inter call interval (ms)	100	80	20	18	10	9
Call duration (ms)	12	7	2	2	1.5	0.75
Terminal frequency (kHz)	26	26	26	26	26	23.5
Chirp bandwidth (kHz)	3	4	5	3	3	3
Call power (dB-SPL)^*^	130	130	110	110	100	100

^*^source level is given at a distance of 0.1 m from the mouth.

We further aimed to determine whether a simple biologically plausible sensorimotor model could explain how bats are able to fly at such high conspecific densities when relying only on active echolocation. We validated the reliability of the model by testing whether it could explain the individual acoustic input recorded onboard the bats. Both the conspecific masking and the echo-jamming proportions predicted by the model represented the trends observed in the real data (red lines in Fig. 2 *B* and *C*). Using the model, we also estimated the expected masking/jamming proportion, which is higher for a real bat, that is more sensitive than our microphone (red dashed line in *SI Appendix*, Fig. S3). After confirming that our model fitted the data, we were able to estimate the proportion of jammed echoes reflected by nearby conspecifics. Nearby conspecifics were defined as conspecifics that flew within the response range (3 m with a 60° double-sided sector, Fig. 2*D*), as this is a reasonable distance and direction for a bat to respond to a nearby object (52). The ability to estimate how many echoes were actually jammed and the expected collision rate is the main advantage of using a sensorimotor model to analyze collective dynamics.

Our model predicted a very low average collision rate of 1.2 ± 0.8 collisions per bat in a group of 2,000 bats during the first 1.3 km of flight (Fig. 2*G*). Notably, the majority of collisions occurred at the cave entrance (note the fast-initial increase in Fig. 2*G*), as we observed in reality (Video S1).

Certain bat species aggregate in huge colonies (47). However, previous research has indicated that even in a colony that is 300 times more populous than our colony (~600,000 vs. 2,000 individuals), the emergence rate is only 4.6 times higher [115 vs. 25 bats per second (47)], with bats emerging over several hours.

**Fig. 3.**
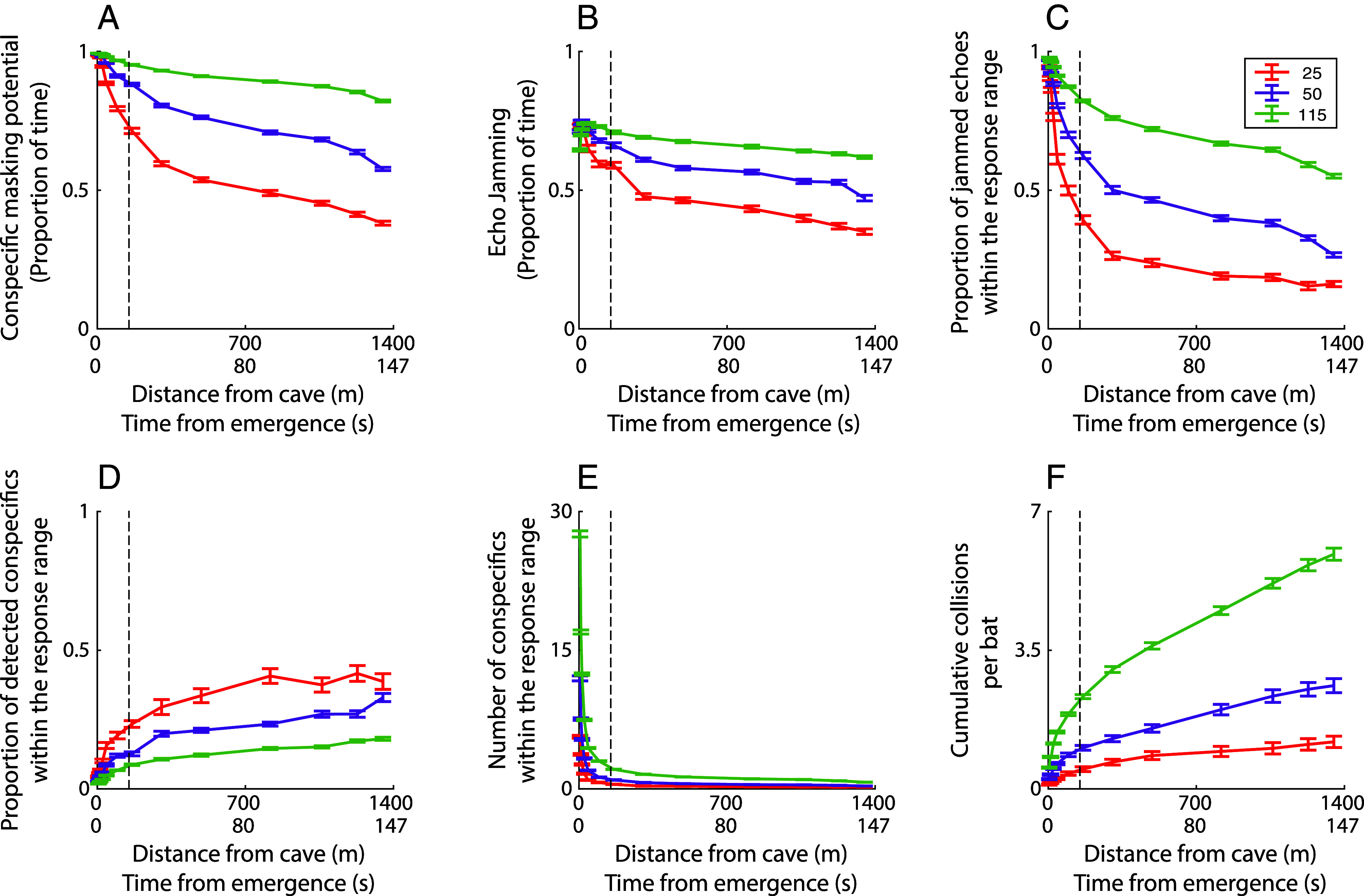
Acoustic masking and collision rate in groups with different emergence rates. In all panels, groups with 25, 50, and 115 bats per second, with a bat hearing threshold of 20 dB (50, 53), are represented by red, purple, and green lines, respectively. Data are presented as a function of the flight distance and time from the cave. Each line represents the mean of 30 simulations and error bars indicate SE. (*A*) Conspecific masking proportion; (*B*) echo-jamming proportion; and (*C*) proportion of echo-jamming out of the total number of echoes (Number of conspecifics) within the response range as a function of time/distance from the cave. (*D*) The proportion of detected conspecifics out of the total number of nearby conspecifics within the response range; and (*E*) the average number of nearby conspecifics within the response range. Nearby conspecifics were defined as conspecifics that flew within the response range, at a distance of 3 m with a 60° double-sided sector relative to the flight direction of the focal bat. (*F*) The cumulative number of collisions per bat. The collision probability decreased dramatically within 150 m from exiting the cave at all emergence rates (see the dashed black line in all panels).

We used our sensorimotor model to examine the bats’ ability to maneuver at higher emergence rates of 50 and 115 bats per second. We found that, at all bat densities, both the conspecific masking and echo-jamming proportions decreased within seconds following emergence (Fig. 3 *A* and *B*). We then examined the proportion of jammed and detected echoes reflected by nearby bats (within the response range). see *Materials and Methods*, Fig. 3 *C*–*E*). Notably, as seen for the lower bat density above (Fig. 1, 25 bats), when the bats move away from the cave, their density declines rapidly and, consequently, the probability of detecting conspecific echoes increases, and the probability of collision decreases (Fig. 3 *D*–*F* and *SI Appendix*, Fig. S4).

To examine the effect of lower densities than the one reported above, we analyzed data from another individual bat that naturally emerged from a different colony with a 25% smaller population and indeed found that the overall masking density is smaller, but the pattern of the quick drop in interference as bats fly away from the cave remains the same (*SI Appendix*, Fig. S5). This also demonstrates the advantage of separating from the main group and flying in smaller groups, as these bats do (41).

**Fig. 4.**
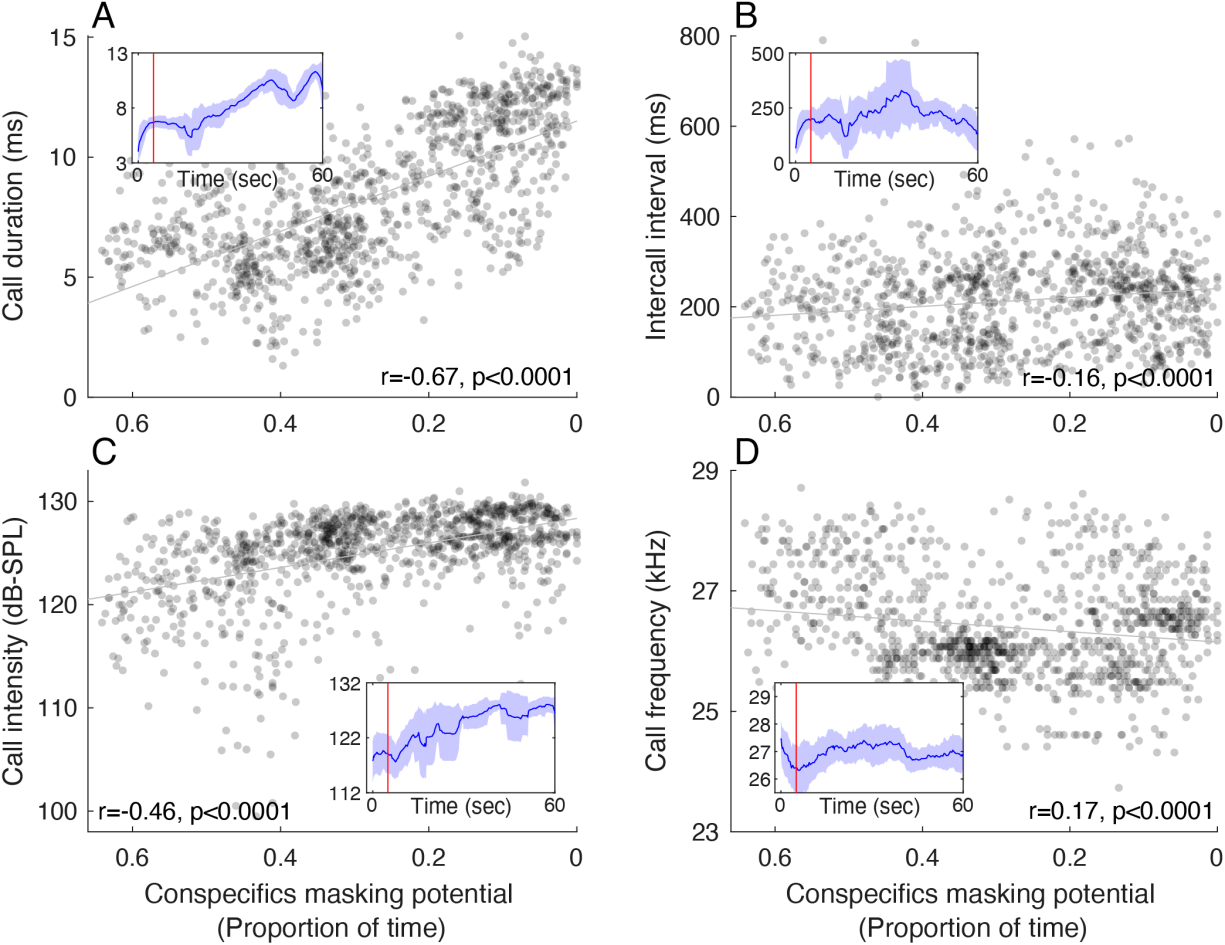
Echolocation characteristics vary with conspecific masking. (*A*) Call duration, (*B*) Intercall interval, (*C*) Call intensity, and (*D*) Call peak fundamental frequency as a function of conspecific masking (the proportion of time with detectible conspecifics). Bats emitted significantly shorter and more frequent calls at a higher frequency and lower intensity as the proportion of conspecific masking increased. Call intensity is presented in dB-SPL and was measured at 0.1 m from the microphone. r and *P* represent the results of Pearson’s correlation tests between each parameter and the masking proportion. GLMM model with conspecific masking as a fixed factor and bat identity as a random effect (*P*-value and the adjusted R^2^ for GLMM, with n = 4 bats for all parameters; call duration: *P* < 0.001 R^2^ = 0.55; intercall interval: *P* < 0.001, R^2^ = 0.04; call intensity: *P* < 0.001, R^2^ = 0.38; call frequency: *P* < 0.001, R^2^ = 0.57 n = 1,068 calls). The insert panels represent an example of the same echolocation characteristics of one bat as a function of time. Note the rapid change in echolocation parameters during the first five seconds (marked by the red lines). The solid lines and shaded area represent a moving average and SD, respectively, with a window of 15 calls.

Finally, we examined how bats adjusted their echolocation in the presence of nearby bats. Echolocation was significantly affected by the bats’ density and conspecific masking (Fig. 4 and *SI Appendix*, Fig. S6). Similar to our previous findings (40), we observed a significant negative relationship between the density of conspecifics (assessed by the conspecific masking proportion) and call duration, intercall interval, and call intensity. A positive relationship was found between the conspecific density and call frequency (which is known to be negatively related to call duration). All correlations were significant [Pearson’s correlation test and generalized linear mixed-effect model (GLMM) with conspecific masking set as a fixed factor and bat identity as a random effect (see *P*-values in Fig. 4)]. These echolocation adjustments are typical of the clutter response in echolocating bats (40, 54–61) which is activated when a bat flies near reflecting objects (such as other bats) and cannot be considered a jamming avoidance response as all bats responded in the same direction (and thus the risk of jamming does not decrease). Had the bats tried to overcome the sensory challenge (rather than avoid obstacles), we would have expected the echolocation adjustments to be in the opposite direction (39, 42, 43).

Another suggested mechanism to avoid masking is spacing the timing of the calls so that the reflected echo will not be masked by the interference, as was observed under lab conditions (36). We found, however, that bats increased their calling repetition rate under higher densities, a behavior that increased the chances of jamming conspecific echoes.

## Discussion

Our study examined how bats contend with the “cocktail party nightmare” (25) they experience during their evening emergence when flying together with thousands of bats in what is arguably one of the most difficult sensory tasks of collective movement. We found that the bats gradually increased their spread as they flew farther from their cave while still maintaining a group structure over several kilometers. This movement strategy allowed the bats to rapidly reduce group density and, consequently, to decrease conspecific sensory masking and almost nullify collision risk.

This type of movement would also benefit bats when searching for ephemeral prey in a group, enabling them to search a larger area (49, 62). However, reducing bat density by spreading out decreases the probability of detecting conspecifics and might thus challenge the group’s cohesion (25). We suggest that to counter this, bats maintain interindividual distances that enable them to move collectively while dramatically reducing their collision probability. In the case of the greater mouse-tailed bat, the large group split up into smaller searching groups.

Our findings also suggest how bats avoid collisions at the very beginning of emergence from the cave when their density is highest. Our sensorimotor model shows that while 94% of the echoes within the response range (~3 m) are jammed at this stage, only 63% of the echoes that are reflected by the closest conspecifics (at a distance of up to 1 m), where collision risk is highest, are jammed (*SI Appendix*, Fig. S7). This is due to the rapid attenuation of echoes (∝1/distance^4^), increasing the probability that echoes from less than 1 m are detected above the masking level. In addition, bats receive multiple echoes from the same conspecific (over consecutive calls), allowing them to use the nonjammed echoes to avoid collisions (63). Our findings suggest that bats may experience up to a maximal rate of 0.8 to 3.6 collisions per minute, respectively, for emergence rates of 25 to 115 bats per second (Fig. 3*F*).

Moreover, even in cases of colliding with conspecifics, our model predicts that bats will mostly maneuver partially before the collision, resulting in lighter impacts. Indeed, bats can easily adjust their flight and recover from such light aerial collisions (64). Our recordings may overestimate sensory interference because real bats have directional pinnae that limit the sector from which interference is received. Moreover, the simulated bats moved in 2D, thus representing the worst-case scenario (they can only alter their horizontal flight direction to avoid collisions), while real bats spread in 3D and can also adjust their altitude. Furthermore, we assume reliance on echolocation alone, while real bats can exploit additional sensory information (see below).

Different solutions have been suggested to overcome the “cocktail nightmare” problem (25, 45, 65). With a few theoretical exceptions (45), most studies focused on small foraging groups and not on bats flying in large groups. Our findings align with an earlier theoretical study that modeled bats’ ability to detect conspecifics in different group sizes, revealing that bats were able to detect their nearest neighbor even in a group of 100 individuals per ~40 m^2^. Therefore, we conclude that the “cocktail party nightmare” might not be as challenging after all (45). Our model improves this previous open-loop model (45), which only modeled sensory detection, by closing the loop and modeling the movement in response to sensory input, using biologically plausible parameters and simple movement control strategies.

Many studies have explored how bats adjust their echolocation in the presence of conspecifics and suggested different solutions for the potential interference (45, 65). Examining bats’ response to severe conspecific masking, we find that bats adjusted their echolocation by decreasing call duration, intercall interval, and call intensity while increasing call frequency according to the typical clutter response which they would execute in the presence of any (animate or inanimate) echo-reflecting object. This response is probably intended to increase ranging accuracy, improve orientation in cluttered environments, avoid signal-echo overlap (66), and increase information rate. The response is not a jamming avoidance response since it will not result in decreased jamming, as previously demonstrated (40, 44).

Our findings revealed that the bats decreased their intercall intervals to improve neighbor detection, contrasting with Beleyur and Goerlitz findings (25, 62), who suggested that longer intercall intervals should improve sensing. Their model suggested an ideal theoretical response for detecting conspecifics. However, in reality, bats must also avoid collision, which requires localization alongside detection. We suspect that these considerations explain the difference between their theoretical results and our real-life observations.

This study focuses on the use of active hearing (i.e., echolocation) for collective movement, though it is possible that bats might employ additional sensory modalities in such situations. Greater mouse-tailed (and many other) bats usually emerge from their cave at twilight, when vision is still useful, possibly considering both modalities when flying in a group (67, 68). However, vision is not always relevant, such as in very large colonies, in which the first individuals emerge in dim light while others emerge in complete darkness, as well as when bats fly inside totally dark caves. Bats might also combine active and passive hearing and sometimes vision to facilitate movement in a group. They can eavesdrop on the bats’ emissions, localize them, and adjust their position within the collective accordingly. This study aimed to determine whether movement at such high densities is possible when using echolocation only and confirmed that it is. By combining data from real bats and modeling, we explain how bats orientate in one of the most challenging sensory environments.

## Materials and Methods

All experiments were conducted according to a permit from Tel-Aviv University IACUC (Number: L-15-045) and the Israel Nature and Parks Authority (Number: 2018/41843, 2019/42193).

### Tracking Bat Collective Movement and Echolocation.

To unravel how thousands of bats avoid collisions with conspecifics when flying under severe echo masking and jamming conditions, we monitored the collective movement and echolocation of greater mouse-tailed bats (*R. microphyllum*) that exited their roost in the Hula Valley in northern Israel during evening emergence.

We tracked the movement of multiple individuals simultaneously using a high-resolution, real-time reverse-GPS system that localizes lightweight tags [ATLAS system, Israel (48)]. To record the echolocation of the bats, we also attached an ultrasonic microphone (Vesper, ASD Ltd, Israel) to some of the ATLAS-tagged bats. Movement data were recorded at 1 to 2 Hz and audio was recorded at a sample rate of 100,000 Hz.

In total, we tagged 110 bats with ATLAS tags and successfully gathered data for 96 of them (59 during 2018 and 37 during 2019). Of these bats, 16 were also tagged with an ultrasonic microphone (Using ATLAS and Vesper tags) to record their echolocation (10 during 2018 and 6 during 2019). In this study, however, we report the body and tag mass of only 14 and 83 tagged bats due to missing data regarding the mass of 19 bats.

The mass of the tags accounted for 3.5 ± 0.6% (n = 83) of the bats’ body mass for ATLAS tags and 11.3 ± 0.9% (n = 14) for ATLAS with Vesper tags [bat body mass: 33.7 ± 4.7 g (n = 104), ATLAS tag mass: 1.1 ± 0.1 g (n = 83), ATLAS with Vesper tag: 4.2 ± 0.2 g (n = 14)]. We have already shown in previous studies that these bats can fly and forage with a GPS device mounted to them that weighs up to 14% of their body mass (40).

The tagged bats were captured a few hours before sunset at the Ein Mimon cave using a hand net. This cave is a summer roost of males, and thus only males were tracked in this study. The bats’ body mass, forearm length, and sex were documented. The tags were attached to the bats using surgical cement (Perma-Type, AC) that lasted for a few days until the tags fell off. The tagged bats were released from a distance of 20 m from the cave into the natural emergence, which emerged at a density of 25 bats per second. Bats were released individually and immediately joined the natural evening emergence that flew above them. The bats were released one-by-one to avoid changing the natural density of the group. Movement data were collected from a distance by the ATLAS system and audio data were collected by recapturing the tagged bats a few days later and recovering the tags.

Altogether, we successfully gathered movement data from all 59 tags in August 2018 and from 37 out of 51 tags in August 2019. In 2018 only two tags were recovered (out of 10 ATLAS with Vesper tags) and provided audio data, and no tags were recovered in 2019 (out of 6 ATLAS with Vesper tags). Additional audio data were collected and analyzed from two more bats from the same cave that were tagged, released near the cave, joined the group, and were recaptured in July 2018 (out of 9 that were tagged in that round).

### Movement Analysis.

The same analyses were performed on the data from both 2018 and 2019 and we present the means of both years. The focal bats’ positions were sampled every 1 to 2 s. However, because not all samples resulted in successful localization, the actual time between successive samples after outlier removal (see below) was 5.8 ± 6.5 s (n = 59) and 2.6 ± 0.6 s (n = 37) for 2018 and 2019, respectively. Bats’ ground speed was calculated according to the time difference and distance between each two adjacent points.

Prior to movement analysis, points with low localization accuracy were excluded from the analysis. Confidence values were assigned as follows: Each point was assigned a confidence value between zero and two according to the number of stations that localized the point and the estimation of the SD of each localization [see more on the accuracy of the ATLAS system in (48, 69)]; points that were localized by seven or more stations or had an accuracy SD of less than 80 were assigned a confidence value of two; points that were localized by three to six stations were assigned a confidence value of one; and all the remaining points were assigned a confidence value of zero. Sequences of five or more consecutive points with a confidence value of one were reassigned to confidence value 2. All points with a confidence value of zero or one were then excluded from the analysis. In addition, outliers were removed if at least one of the following criteria was met: SD of localization quality above 40, ground speed above 20 m/s; and/or flight distance of >500 m from at least one of the five points prior to the examined point. The data were then smoothed using a “loess” local regression smoothing filter (70), using nine data points to calculate the smoothed value and linearly interpolated to once every second.

Bat trajectories were divided into commute and foraging according to a straightness index (71) that measures the ratio between the length of the beeline and the actual flight path (values 0 to 1). The straightness index of each point was calculated according to a flight segment of 60 s (30 s before and 30 s after the specific point or less for the beginning of the night). Points with a straightness index below a threshold of 0.7 were considered as foraging, and points with a straightness index equal to or above this threshold were considered as commute (*SI Appendix*, Fig. S8).

Movement analysis was conducted for commute data of the first ten minutes of flight for each bat. Bat flight trajectories were scrutinized manually, and the bats that flew in a different direction to that of the main group were excluded from the analysis (Fig. 1*B* and *SI Appendix*, Fig. S1*A*). To identify the region covered by the group, we generated 2D probability distributions of the locations of all bats (over all times) with a 50x50 m^2^ bin size. The histogram was then smoothed using a 5-pixel 2D Gaussian kernel. A contour plot was used to determine the outer line of the region of the group and to exclude flight trajectories of bats from the moment they left the group (the borders of the group were defined as the region where the probability of finding a bat was higher than 0.05%).

To estimate the group center, we tracked the ridge with the highest density of bats in the 2D histogram (Fig. 1*A* and *SI Appendix*, Fig. S1*B*). We then measured the average perpendicular distance of each bat from the group center as a function of its distance from the cave, using 100-meter bins (up to 1,400 m from the cave, where the main group split into several directions. Note that we first examined group behavior during the first ten minutes and then analyzed only the first 1,400 m, i.e., the first ~2.5 min).

### Audio Analysis.

Bat echolocation calls were recorded continuously using an ultrasonic microphone affixed to the back of the bats (Vesper, ASD Ltd, Israel; Onboard microphone: SPU0410LR5H-QB, Knowles Electronics LLC; sample rate 100,000 Hz, 8 bit). Audio data were first filtered for audio analysis between 20 to 30 kHz (Butterworth, order = 10), and echolocation calls of the tagged bat and its conspecifics were then scrutinized manually using our in-house MATLAB-based software (Batalef). The analysis encompassed call duration, intercall interval, intensity, and the fundamental frequency of the second harmonic of each call [see (40) for exact acoustic definitions]. To identify the calls of the focal bat, we primarily relied on the intensity of the calls, mainly using the intensity of the first harmonic, which is characterized in this species by lower intensity compared to the second harmonic and was visible mostly only for the focal bat, and thus allowed us an accurate recognition of the calls of the focal bat while flying. When bats were very close to each other, and the intensity of their calls was similar, we also used the expected consistency of the interval between the calls to identify the calls of the focal bat.

The intensity of the calling bat was estimated by calibrating the Vesper microphone to a calibrated GRAS 40DP 1/8inch microphone (GRAS Sound & Vibration) for the relevant frequencies at a distance of 0.1 m. We first converted the intensity that was measured in dB to dB-SPL by adding a value of 139 dB to the intensity of all calls. This value was estimated as the difference between the noise-floor of our microphone (Average of 45 dB-SPL for frequencies of 20 to 30 kHz) and the calls with the lowest intensity calls that were recorded (−94 dB).

We then corrected the intensity of the loudest (and long, >5 ms) recorded calls (106.1 ± 1.4 dB-SPL, n = 2,306 calls), which were slightly clipped due to our microphone’s upper threshold. To correct for the intensity of these clipped calls, we found the fitting curve that characterizes the relationship between call duration and call intensity of nonclipped calls (97.0 ± 5.3 dB, n = 315 calls, $I=0.009x^{3}-0.296x^{2}+3.6x+85.95$, adjusted r^2^ = 0.42, *SI Appendix*, Fig. S9). We then calculated the expected increase in call intensity according to the inclination of the fitting curve of nonclipped calls that were longer than 5 ms, and added this value to the intensity of the clipped calls. Note that this procedure was conducted to correct the clipped calls of both the focal bat and its conspecifics and resulted in an average increase of 2.3 ± 1.3 dB in call intensity (n = 2,306).

Finally, we used the same calibrated GRAS microphones to measure the reduction in call intensity between the mouth of the bat and the location of the microphone on the back of the bat. To this end, we recorded the sound intensity of a hand-held bat on-axis and 180°—at the back of the bat. We found a reduction of ~22 dB between the intensity of the emitted call and the recorded call. To correct for this intensity reduction, we added 22 dB to the measured intensity of the focal bat (*SI Appendix*, Fig. S10).

### Conspecific Masking Potential.

The conspecific masking proportion was calculated in time windows of one second as the proportion of time that the frequency band of the second harmonic (26.4 ± 0.7 kHz, which carries most energy) of these bats’ echolocation calls (n = 1,923 calls) included detectable conspecific calls (at any sound level, i.e., louder than 45 dB SPL, which is the noise floor of our system, n = 16,432 analyzed conspecifics calls; Fig. 2*A* and *SI Appendix*, Fig. S2). This threshold was applied to the sensorimotor model to equalize conditions.

### Echo-Jamming.

To estimate the potential echo jamming proportion, we calculated the proportion of time out of the total echo-reception window that the intensity of conspecifics’ calls was stronger than the echoes the focal bat was expected to receive from nearby conspecifics (see schematic in Fig. 2*A*). The echo-reception window is the time period after each emission when the bat should receive echoes from nearby neighbors, which are crucial for collision avoidance. Under such conditions, the bat might not detect the echoes reflected from conspecifics and might suffer from actual information loss that could lead to collisions. Due to the noise-floor of our microphone, in most cases, we could not detect the reflected echo, and therefore we estimated the level of an echo reflected by a hypothetic conspecific. We took into account the nearest conspecific located in front of the bat (within its emitted beam-width, at a maximum distance of 3 m ahead). We did this for different distances from the cave, with the bats distributed at each distance according to the estimated bat density. The reflected echo of an echolocation call when reflected from a small approximately spherical object like a bat is a delayed attenuated version of the call. Thus, we defined the potential echo window as having the same duration as the call (8.3 ± 1.4 ms, n = 4 bats). The time when the reflected echo arrives back to the bat depends on the speed of sound and the distance between the bat and the reflected object. We estimated the time of echo arrival 28.7 ± 7.2 ms, n = 4 bats) according to the two-way time travel of sound from the focal bat to its nearest neighbor located at most 3 m away (the reaction radius). We refer to this window as the conspecific echo-reception-window. We estimated the expected intensity of conspecific echoes by calculating the geometric and atmospheric attenuations (55) according to the sonar equation (52) and the target strength of a bat (as described in greater detail in the Sensorimotor model below).

Note that our definition of jamming does not assume a biological receiver, which sometimes enables bats to detect echoes even in a negative signal-to-noise ratio (72). This analysis only sought to reveal the rapid decrease in jamming over time, while our model, presented below, already incorporates a more sensitive receiver.

In addition to estimating the potential echo jamming proportion (the proportion of time that echoes from the nearest neighbor could be jammed), our sensorimotor model allowed us to estimate the proportion of echoes that were actually jammed, which depends not only on the masking signal but also on the arrival time of an echo. This could be estimated using our model but could not be measured directly for the real bats, because conspecific echoes are usually too weak to be detected by our tags. The ability to estimate how many echoes were actually jammed and the expected collision rate is the main advantage of using a sensorimotor model to analyze collective dynamics.

### Sensorimotor Model.

The sensorimotor model is described in detail in (44), and is described here briefly, highlighting our modifications. The model comprises multiple bats flying and echolocating in an open two-dimensional area. Each bat transmits echolocation calls and receives echoes reflected from nearby objects, as well as conspecifics’ calls, which may mask the desired echoes.

The models’ parameters were taken to represent greater mouse-tailed bats’ movement and echolocation while emerging from a cave. We set the number of bats in the model to 25 bats per second, ran the simulation for 50 bats placed on a two-fold larger plane (i.e. while increasing only the x-axis), and analyzed the results for the 25 bats in the middle to avoid edge effects (Fig. 2*D*). The initial position of the bats in the axis orthogonal to the group’s movement direction (i.e. y-axis) was randomly distributed with a mean of zero and a SD of 11°, fitting our measurements of the real group as a function of the distance from the cave (Fig. 1*C*). The plane length in the direction of the group’s flight (i.e., the x-axis) was set to 3 m. This value represents the distance between the first bat and the last bat during one second, based on the estimated bats’ density and speed in our video recording (Video S1). It was converted from the 3D group to our 2D model, thus representing a more severe scenario. To estimate the interindividual distances, we generated random positions of the bats according to a normal distribution with a SD based on the measured positions of the real bats from the center of the group. We calculated the mean minimum distance between each pair and repeated this process 30 times. Additionally, we tested the model with various numbers of individuals (50 and 115 bats, Fig. 3), as well as with a group twice as dense as the real bats, while they are spread on a plane with a maximal distance in the direction of the group’s flight of 1.5 m (*SI Appendix*, Fig. S4).

The simulated bats start flying in a random direction, as described above. They keep flying in a “correlated random walk” (73) with a gradually increased velocity and then remain flying at a constant velocity (as the real bats did) as long as they do not get too close to conspecifics (3 m). In such situations, the bats change their flight direction to the opposite direction of their nearest conspecific and adjust their velocity, trying to avoid collision. The bats return to a correlated walk flight path after this avoidance maneuver ends, i.e., when there are no more detected conspecifics within 3 m in front of them. Note that, theoretically, a bat can detect a conspecific using echolocation from a distance of 5 m away [according to the sonar equation (52), taking into account the echolocation characteristics in Table 1 and a hearing threshold of 20 dB-SPL (50)]. However, it is very unlikely that a real bat will collide with another bat so far away, and thus, the simulated bats maneuver only when detecting a conspecific within 3 m. This was also confirmed with a sensitivity analysis (*SI Appendix*, Fig. S7).

The bats in the simulation emit Frequency Modulated (FM) down-sweep calls according to the acoustic behavior of greater mouse-tailed bats (their second harmonic; see Table 1). The echolocation behavior of the simulated bats is divided into three main phases: “Search,” “Approach,” and “Buzz” (50, 74, 75). The phase and the transitions between phases are determined by the distance to the nearest detected object (i.e., other bats, in our scenario): “Search” switches to “Approach” at 3 m from the target, “Approach” switches to Buzz’ at 0.4 m, and “Terminal Buzz 1” changes to “Terminal Buzz 2” at 0.2 m from the target. During each phase, the intercall interval, call duration, bandwidth, and call power (in dB) are reduced linearly between the start and end values (Table 1).

When the simulated bat detects conspecifics, it estimates their distances and directions based on an analysis of the complete auditory scene, including the echoes and masking calls. The distance is determined by the time of the correlation’s peak, and the direction of the echoes consists of a random error with a SD based on the signal to interference ratio (44). Using a correlation detector (44) echoes from other bats will only be processed if they cross an auditory threshold [set to 20 dB-SPL (50)] and if they are not masked by conspecifics’ calls (44). The levels of the reflected echoes were calculated according to the sonar equation [equation 5.36, pp. 181 (52)]. The model accounts for the distance and direction to the target, the directivity of the ears and mouth, and the atmospheric absorption (44). The target bat is modeled as a disc with a radius equal to 15 cm (which resembles the wingspan of this species, forearm 7.0 ± 0.7 cm, n = 97), equally reflecting in all directions (the target strength of a disc with a radius of 0.15 m is 22.5 dB).

For each bat, we calculated the conspecific masking and echo-jamming potentials as a proportion of time, as described above (see *Audio Analysis* in *Materials and Methods*). However, here we first excluded all calls weaker than 45 dB SPL, according to the recording noise-threshold of our ultrasonic microphone. We then used a similar approach but excluded all calls weaker than 20 dB SPL to estimate conspecific masking and echo-jamming potentials according to actual bats’ hearing threshold (50).

For the simulated data, we also calculated the echo-jamming rate within the response range (3 m with a 60° double-sided sector), which is defined as the ratio between the number of jammed echoes and the total number of received echoes (jammed echoes were identified using a correlation detector). This could not be done for the real bats as the echoes were mostly undetectable. The received echoes comprised all echoes whose received intensity level was above the detection threshold. Jammed echoes are received echoes that were masked by the louder conspecific calls. We defined a collision as the event of bats getting within 10 centimeters of a conspecific.

### Statistical Analysis.

Statistical analysis, acoustic simulation, and movement simulation were conducted using MATLAB R2022a with a significance level of 0.05. Movement analysis was conducted using MATLAB R2022a, except for cleaning the data, which was performed using R version 2022.07.0.

Movement analysis was conducted for 59 and 35 bats in 2018 and 2019, respectively, and acoustic analysis was conducted for four bats. Movement and acoustic simulations were conducted on 30 trials per condition. All statistical tests are described throughout the text, together with the statistical test we used and the relevant sample size.

## Supplementary Material

Appendix 01 (PDF)

Movie S1. Evening emergence of greater mouse-tailed bats (*Rhinopoma microphyllum*)

Movie S2. An illustration of the sensorimotor model. In the simulation, 25 bats fly for a second at a distance of 40 m from the cave, emit echolocation, and respond according to their reflected echo to avoid collisions. The intensity of the echolocation of the focal bat (black) and other conspecifics (orange), as well as the echoes of the focal bat (green), are presented in the lower panel. The acoustic scene is presented from the point of view of the focal bat; the conspecifics it detects are marked with green stars, and its jammed echoes are marked with red diamonds. The movie is presented at a speed 18 times slower than in real-time.

## Data Availability

Movement and audio data have been deposited in Mendeley (https://doi.org/10.17632/k7pt2j84f7.1) (76). All study data are included in the article and/or supporting information.
