# Supplementary material for: Onboard recordings reveal how bats maneuver under severe acoustic interference
Source: Proc Natl Acad Sci U S A. 2025 Mar 31;122(14):e2407810122. doi: 10.1073/pnas.2407810122 (PMC12002023; doi:10.1073/pnas.2407810122)
Supplement: Supplementary file 1 — Appendix 01 (PDF) [file pnas.2407810122.sapp.pdf]

PNAS

Supporting Information for

On-Board Recordings Reveal How Bats Maneuver Under Severe Acoustic Interference

Aya Goldshtein[1,2,3,4†*], Omer Mazar[5†], Lee Harten[4], Eran Amichai[4], Reut Assa[4], Anat Levi[6,7], Yotam Orchan[6,7], Sivan Toledo[7,8], Ran Nathan[6,7], & Yossi Yovel[4,5,9*]

† These authors contributed equally

*Corresponding authors: Aya Goldshtein and Yossi Yovel
Email: agoldshtein@ab.mpg.de or goldaya@gmail.com, yossiyovel@gmail.com

This PDF file includes:

Figures S1 to S10
Legends for Videos S1 to S2

Other supporting materials for this manuscript include the following:

Videos S1 to S2

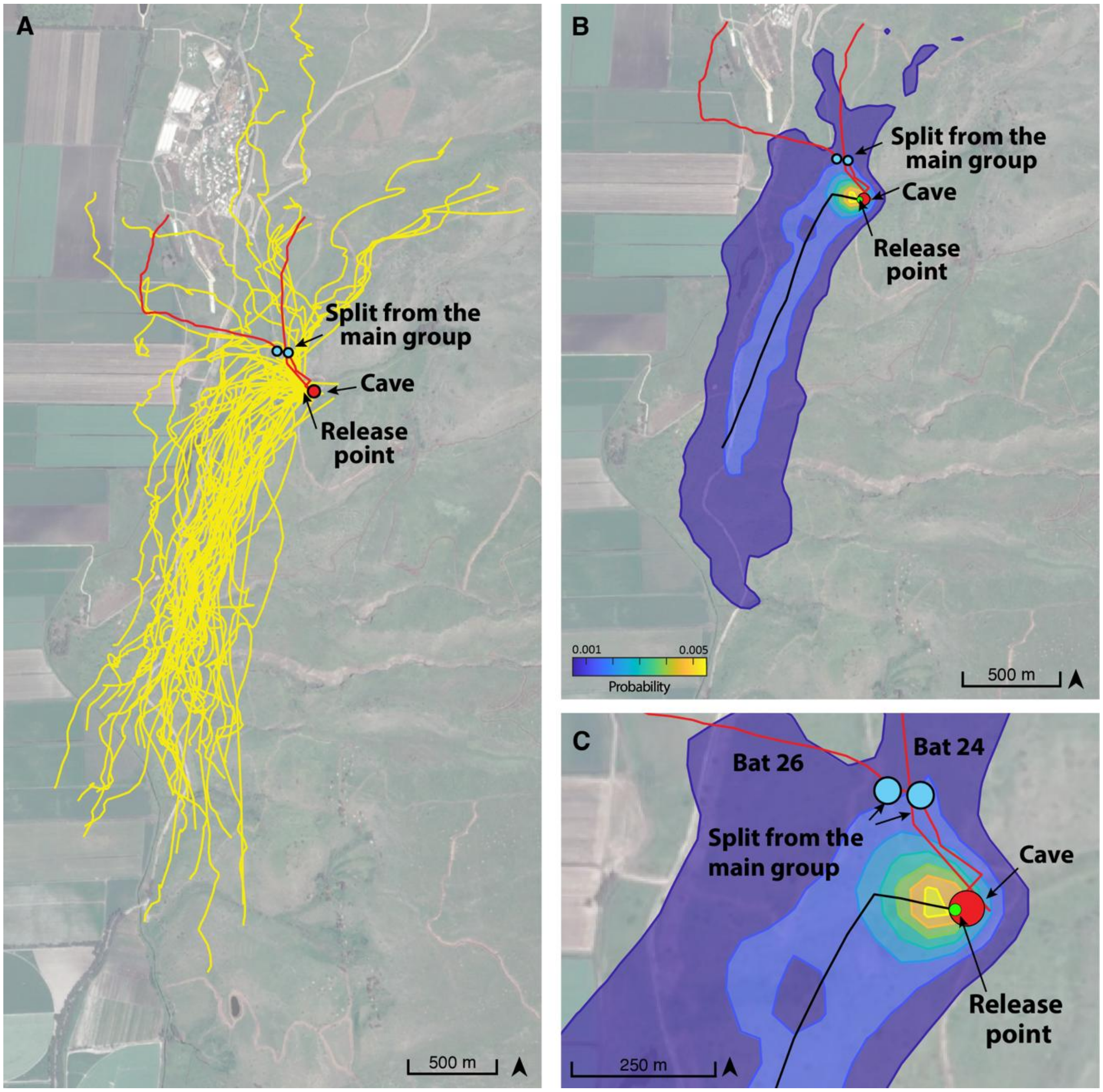

Fig. S1. Bats' trajectories and density during evening emergence in 2018. Tagged bats were released at a distance of 20 m from the cave, one by one, and immediately joined the natural evening emergence that flew above them to avoid changing the natural density of the group (the cave is represented by a red circle). (**A**) Most bats flew in the main group during the 1.5 km (72% out of 57), while others flew in different directions and were excluded from data analysis. Audio recordings of bats 24 and 26 (red lines) were used for audio analysis. Note that these bats flew within the main group during the first 170 m, bat 26 flew another 450 m at the edge of the main group, and bat 24 splits from the main group and fly together with other bats (28% of the tagged bats) for additional 300m (the points where they split from the main group is represented by light blue circles). Data represents bats' trajectories during the first 2.5 minutes after the release. (**B**) Probability of bat density. The colored contour lines represent the normalized density, from low density in blue to high density in yellow. The black line represents the center of the main group. (**C**) Zoom in on the flight trajectories near the cave, emphasizing that bats 24 and 26 flew in the main group at the first 170 m from the cave. The curve that is shown for bat 26 is a result of a tracking outlier (indicated by significantly high flight speed >20 m/s).

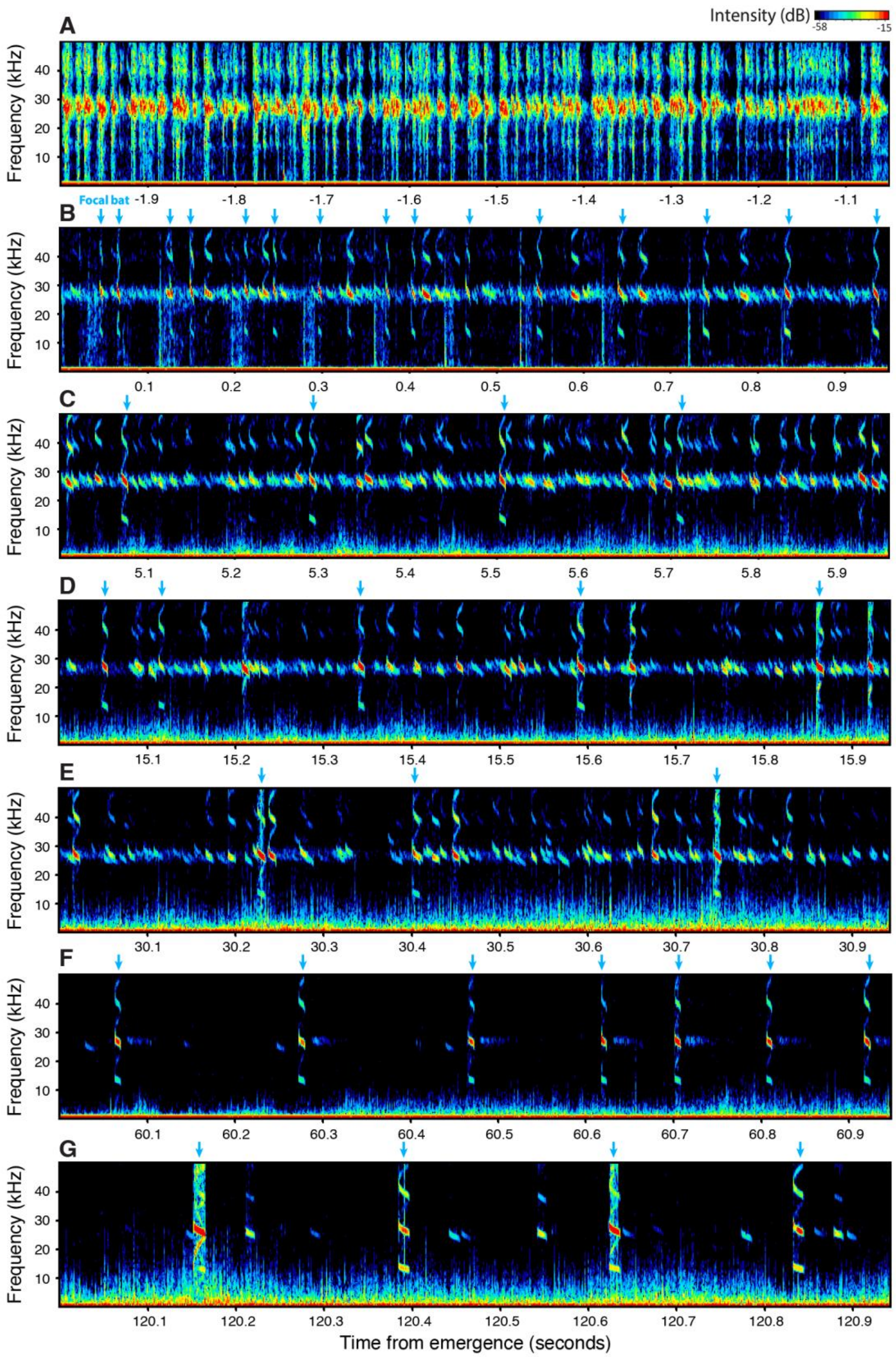

Fig. S2. The acoustic scene within the group from a single bat point of view at different times from emergence. The acoustic scene was recorded by one of the tags attached to a bat at various times, from two seconds before emergence to 120 seconds after emergence. Focal calls are marked with blue arrows.

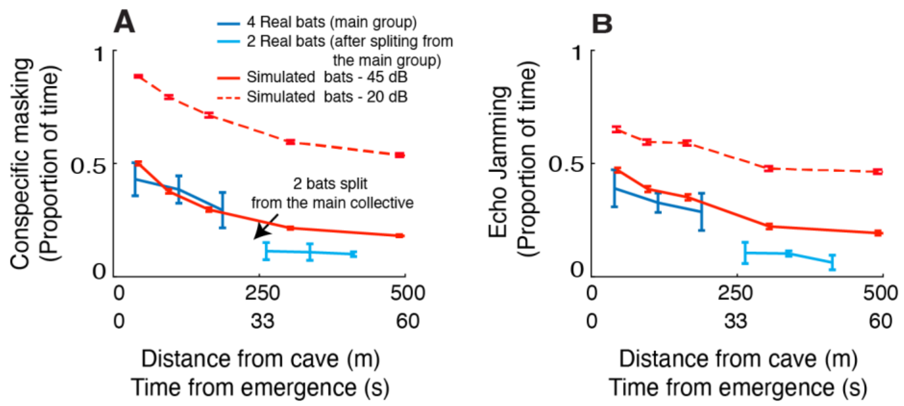

Fig. S3. Masking levels during bat emergence. (**A-B**) Echolocation masking and jamming proportion as a function of flight distance or time from the cave (data represents mean and standard error for four/two real bats (dark/light blue) and for 30 simulations for the simulated bats (red). Data for four bats that fly within the main collective are depicted in blue (25-200 m from the cave), and data for 2 bats that split from the main collective and fly with a smaller group are depicted in light blue (200-450 m from the cave). The red lines in A-B show the masking-jamming proportions for simulated bats when using a hearing threshold of 45 dB-SPL (the noise floor of our microphone, solid lines) and when using a hearing threshold of 20 dB-SPL [the hearing threshold of real bats (74), dashed lines].

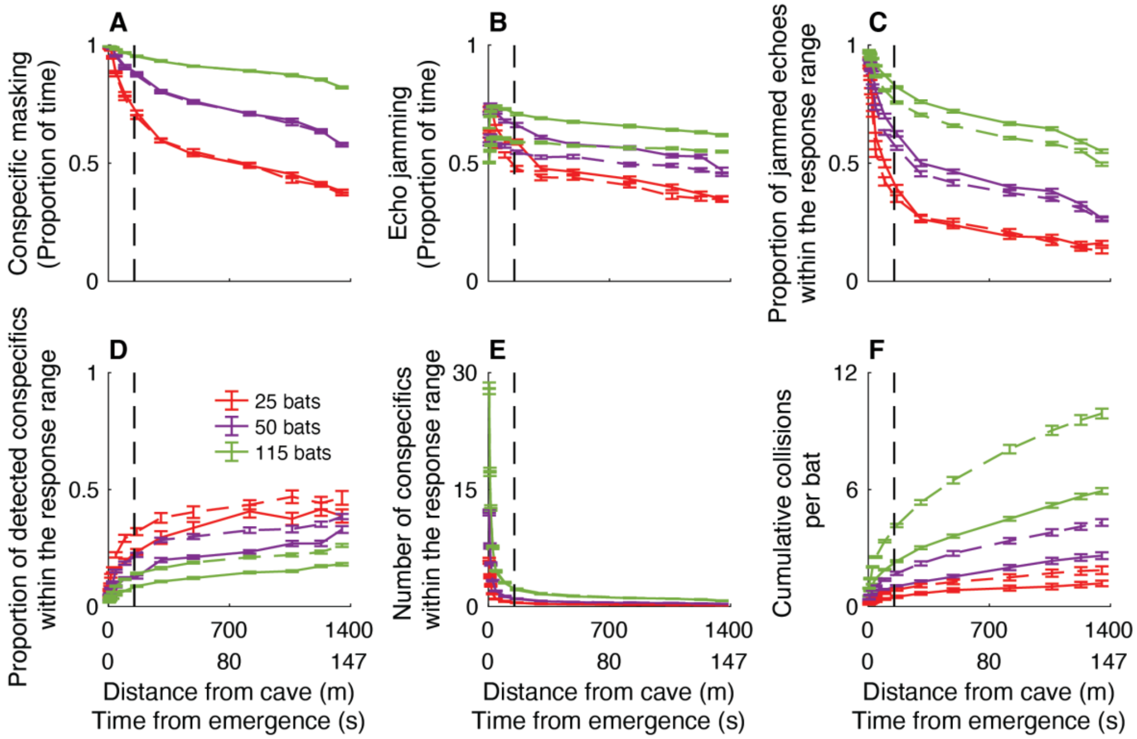

Fig. S4. Acoustic masking and collision rate in modeled bats with different emergence rates and densities in the sensorimotor model. In all panels, groups with 25, 50, and 115 bats per second and a hearing threshold of 20 dB are represented by red, purple, and green lines, respectively. We tested two spatial densities for each temporal density: the low density (3 m x-axis), which was estimated by the real bats, is represented by solid lines, and the double density (1.5 m x-axis) is represented by a dashed line. Data is presented as a function of the flight distance and time from the cave. Each line represents the mean of 30 simulations, while error bars indicate standard errors. (**A**) Conspecific masking proportion, (**B**) echo jamming proportion, and (**C**) proportion of echo jamming within the detection range as a function of time/distance from the cave. (**D**) The proportion of detected conspecifics out of the total number of nearby conspecifics with whom collision is relevant and (**E**) the average number of nearby conspecifics within the response range. Nearby conspecifics were defined as conspecifics that flew within the response range at a distance of 3 m with a 60-degree double-sided sector relative to the flight direction of the focal bat. (**F**) The cumulative number of collisions per bat.

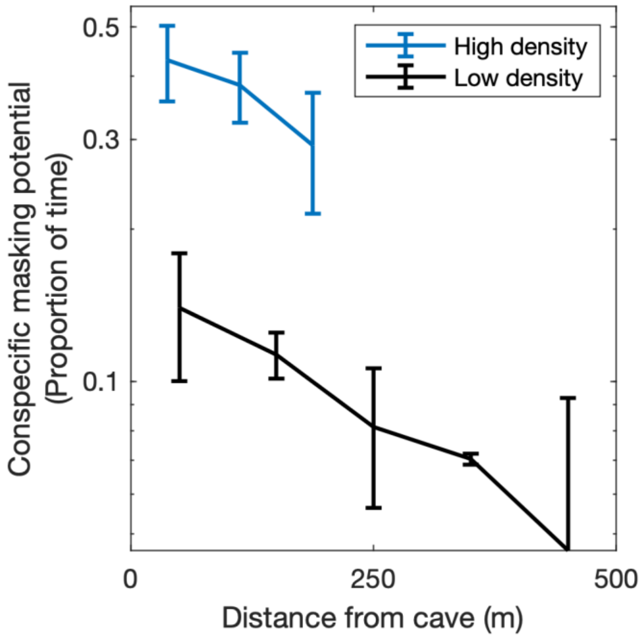

Fig. S5. Acoustic masking in a lower density colony of ~1500 bats during natural evening emergence. The decrease in conspecifics masking potential, as presented for our real bats (blue, high density, ~2000 bats), is also observed in the lower density when bats naturally emerged from their cave (black, low density, 1500 bats). Data represents mean and standard error, and the y-axis is represented on a log scale.

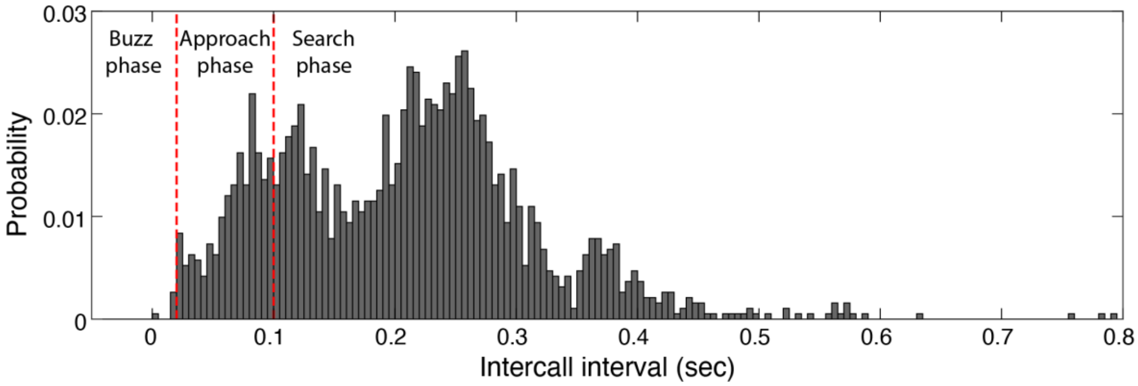

Fig. S6. Probability of intercall interval during bats' emergence from their cave. During the initial emergence, bats emitted approach calls and search calls, while buzz calls were hardly exhibited (see Table 1 for the definition of the different phases).

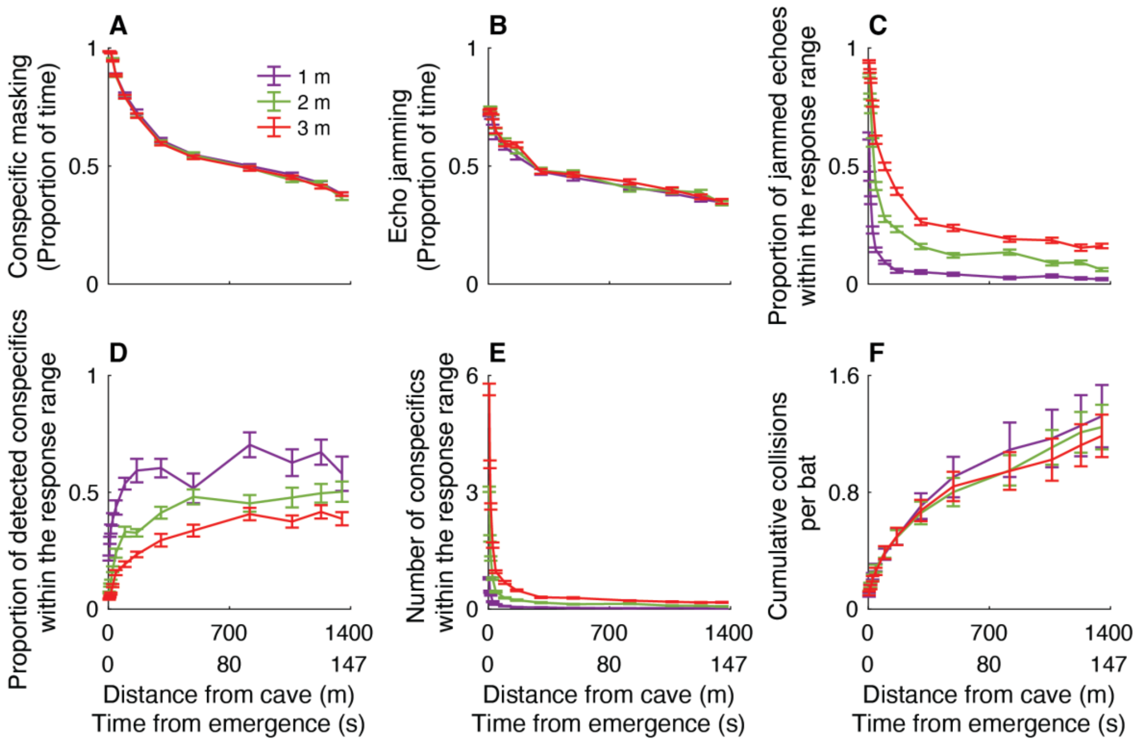

Fig. S7. Acoustic masking and collision rate in modeled bats with different detection distances in the sensorimotor model. In all panels, response distances of 1, 2, or 3 m in a group with 25 bats per second and a hearing threshold of 20 dB, are represented by purple, green, and red lines, respectively. Data is presented as a function of the flight distance and time from the cave. Each line represents the mean of 30 simulations, while error bars indicate standard errors. (**A**) Conspecific masking proportion, (**B**) echo jamming proportion, and (**C**) proportion of echo jamming within the response range as a function of time/distance from the cave. (**D**) The proportion of detected conspecifics out of the total number of nearby conspecifics within the response range and (E) the average number of nearby conspecifics within the response range. Nearby conspecifics were defined as conspecifics that flew within the response range at distances of 1, 2, or 3 m ahead and within ±60° relative to the flight direction of the focal bat. (**F**) The cumulative number of collisions per bat.

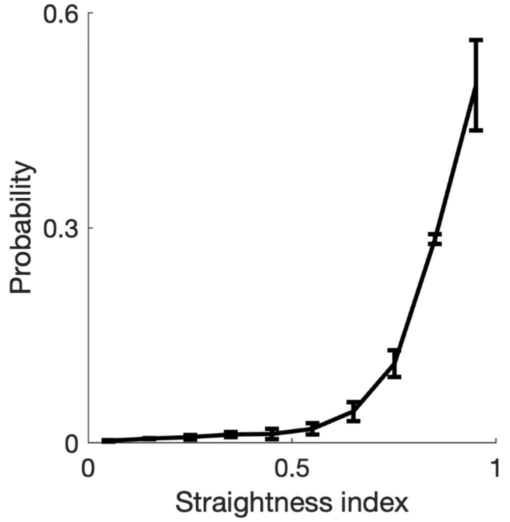

Fig. S8. Bats' movement segmentation to commute and foraging. The distribution of movement straightness index (Mean±SE, n=2 years). A bat's movement was considered as commute when its straightness index was higher than 0.7 and as foraging when its straightness index was lower than 0.7 (this value was chosen as it represents the inflection point in the plot). Note that the analyzed data represents the first 2.5 minutes of flight when bats emerge from the cave and fly in a collective structure, and therefore, most GPS points were considered commutes.

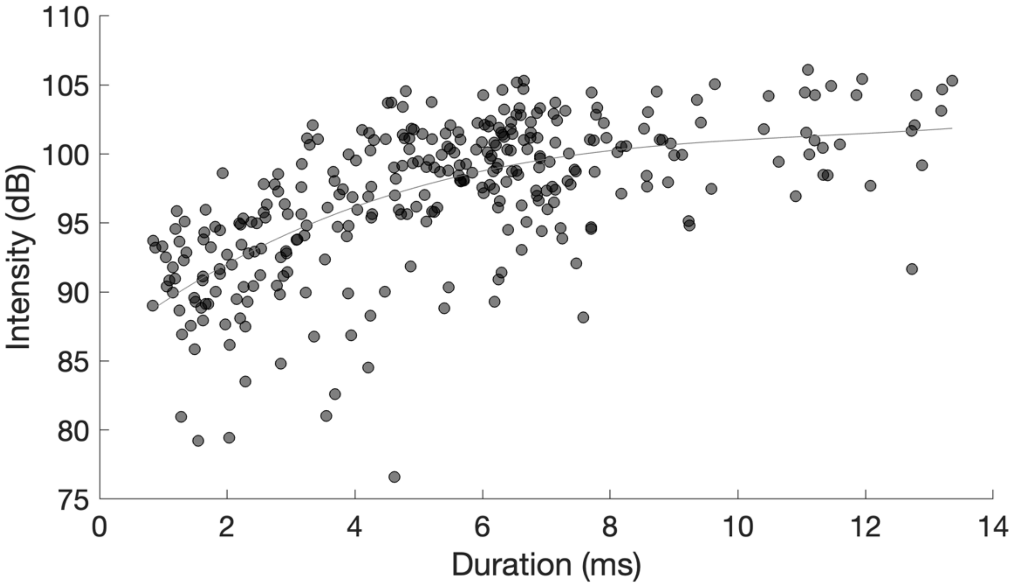

Fig. S9. Call intensity as a function of call duration. The call intensity of non-clipped calls of four bats appears in black dots, and the fitting curve appears as a grey line (97.0 ± 5.3 dB, n=315 calls, $I = 0.009x^3 - 0.296x^2 + 3.6x + 85.95$, Adjusted r^2 = 0.42).

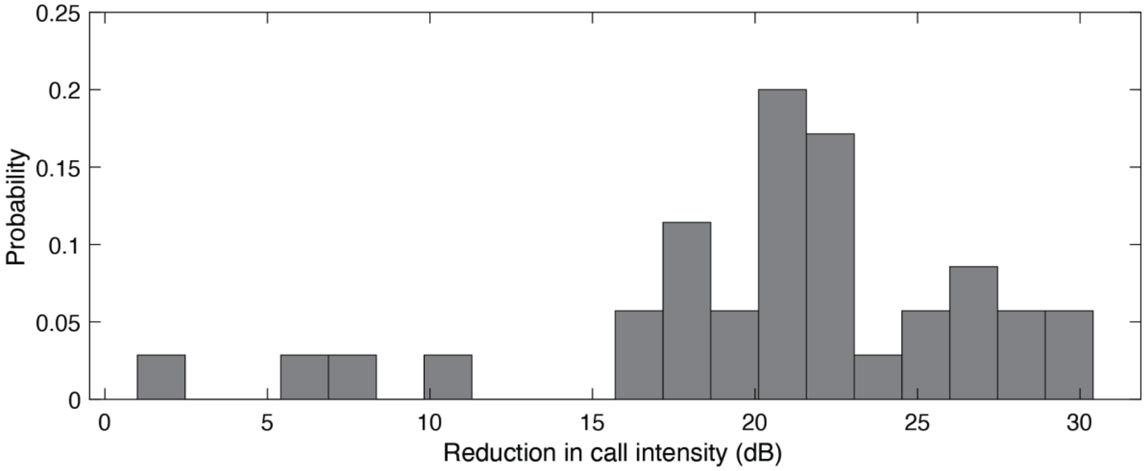

Fig. S10. The reduction in call intensity between the mouth of the bat and the location of the microphone on the back of the bat. We used a calibrated GRAS microphone to measure the reduction in call intensity between the mouth of the bat and the location of the microphone on the back of the bat. We recorded the sound intensity of a hand-held bat on-axis and 180 degrees – at the back and found a reduction of ~22 dB between the intensity of the emitted call and the recorded call.

Video S1 (separate file). Evening emergence of greater mouse-tailed bats (*Rhinopoma microphyllum*)

Video S2 (separate file). An illustration of the sensorimotor model. In the simulation, 25 bats fly for a second at a distance of 40 m from the cave, emit echolocation, and respond according to their reflected echo to avoid collisions. The intensity of the echolocation of the focal bat (black) and other conspecifics (orange), as well as the echoes of the focal bat (green), are presented in the lower panel. The acoustic scene is presented from the point of view of the focal bat; the conspecifics it detects are marked with green stars, and its jammed echoes are marked with red diamonds. The movie is presented at a speed 18 times slower than in real-time.
